# Supplementary material for: Mechanism of sensor kinase CitA transmembrane signaling
Source: Nat Commun. 2025 Jan 22;16:935. doi: 10.1038/s41467-024-55671-3 (PMC11754779; doi:10.1038/s41467-024-55671-3)
Supplement: Supplementary file 1 — Supplementary Information [file 41467_2024_55671_MOESM1_ESM.pdf]

Supplementary Information for

Mechanism of sensor kinase CitA transmembrane signaling

Xizhou Cecily Zhang[1], Kai Xue[1], Michele Salvi[1], Benjamin Schomburg[1], Jonas Mehrens[1], Karin Giller[1], Marius Stopp[2], Siegfried Weisenburger[3,4], Daniel Böning[3,4], Vahid Sandoghdar[3,4], Gottfried Unden[2], Stefan Becker[1,*], Loren B. Andreas[1,*], Christian Griesinger[1,*]

[1]Department of NMR-based Structural Biology, Max Planck Institute for Multidisciplinary Sciences; Am Faßberg 11, 37077 Göttingen, Germany.

[2]Institute for Molecular Physiology (imP), Microbiology and Biotechnology, Johannes Gutenberg University; 55128 Mainz, Germany

[3]Department of Physics, Friedrich Alexander University (FAU) Erlangen-Nürnberg; Erlangen, Germany

[4]Department Nano-Optics, Plasmonics and Biophotonics, Max Planck Institute for the Science of Light, Erlangen, Germany

*Corresponding authors: sabe@mpinat.mpg.de; land@mpinat.mpg.de; cigr@mpinat.mpg.de

This PDF file includes:

Supplementary Figures 1 to 15
Supplementary Tables 1 to 12

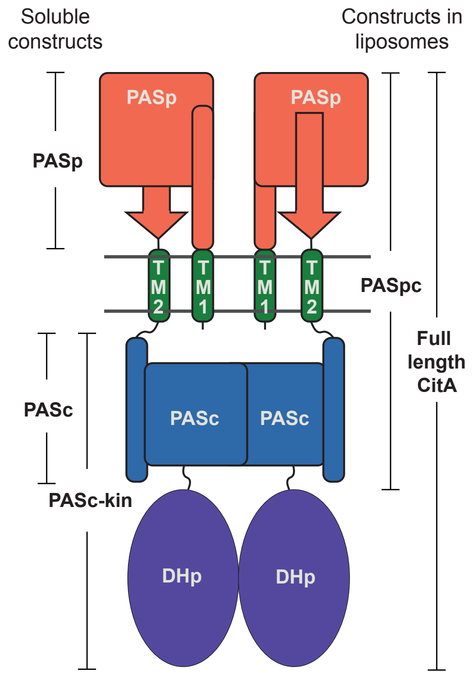

Supplementary Figure 1: Domain architecture of Gt CitA and subdivisions of it that form the constructs used in the work. The soluble constructs are isolated PASp, isolated PASc and PASc-kin which contains the PASc domain and the DHp domain. The solid constructs are the full lenght CitA and the PASpc construct which contains the PASp domain, TM1 and TM2 helices, and the PASc domain.

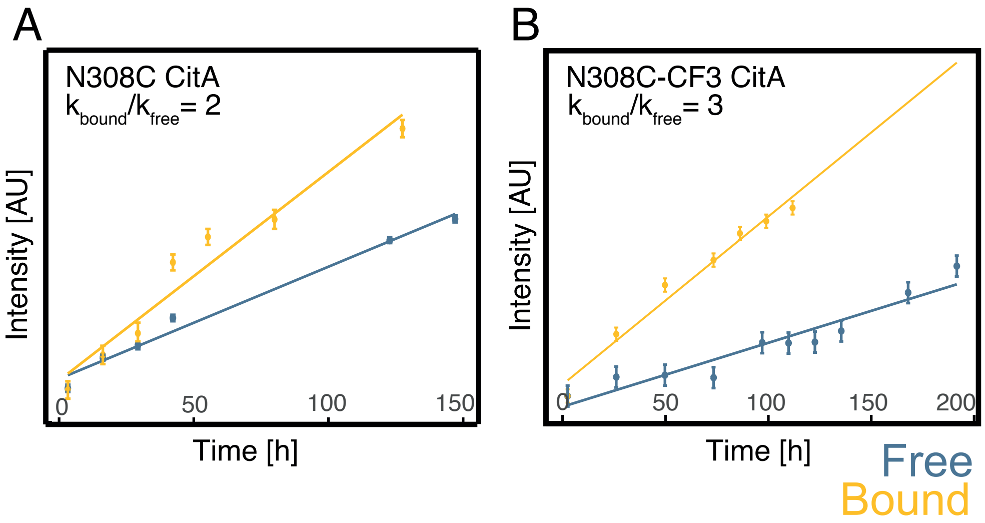

Supplementary Figure 2. Kinase activity of full length C12A/R93A/N308C CitA without tag (A) and with CF3 (B) from ^{31}P NMR assay. The citrate bound state (yellow) in the non-tagged and CF_3-tagged CitA has two to three times higher activity compared with the citrate free state (blue).

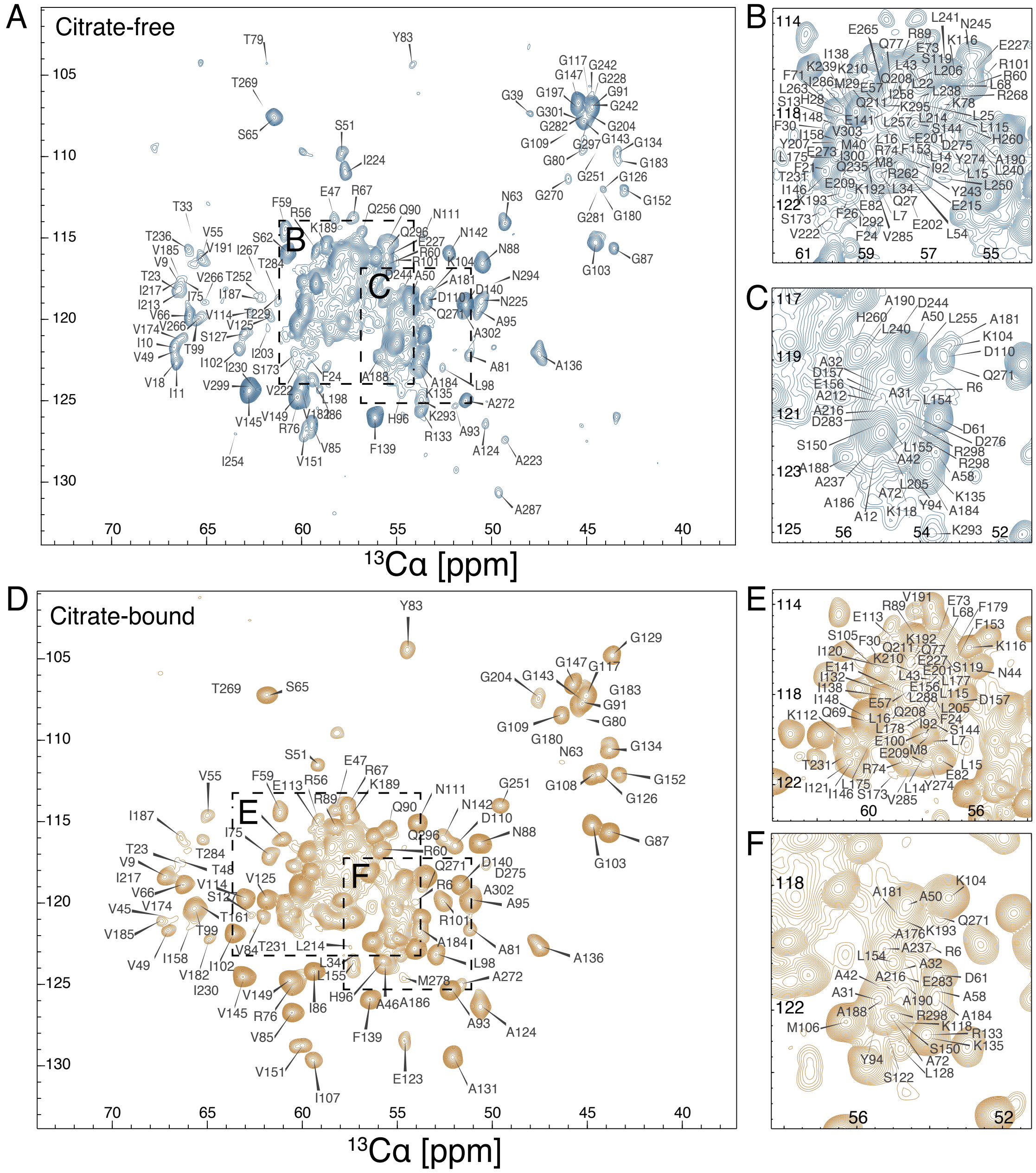

Supplementary Figure 3. Spectra of bound and free lipid-embedded CitApc and residue specific assignment. (A to C) NCα projection of the (H)CANH spectrum in the free state, and (D to F) in the citrate bound state. The assigned chemical shifts are mapped onto the spectra projections. The citrate bound state has more well-resolved peaks than the citrate free state. More assignments were found for the PASp domain in the citrate bound state, while the PASc domain assignments were more extensive in the citrate free state. All resonances from the assignment of the two state are included in Supplementary Datafiles 1 and 2.

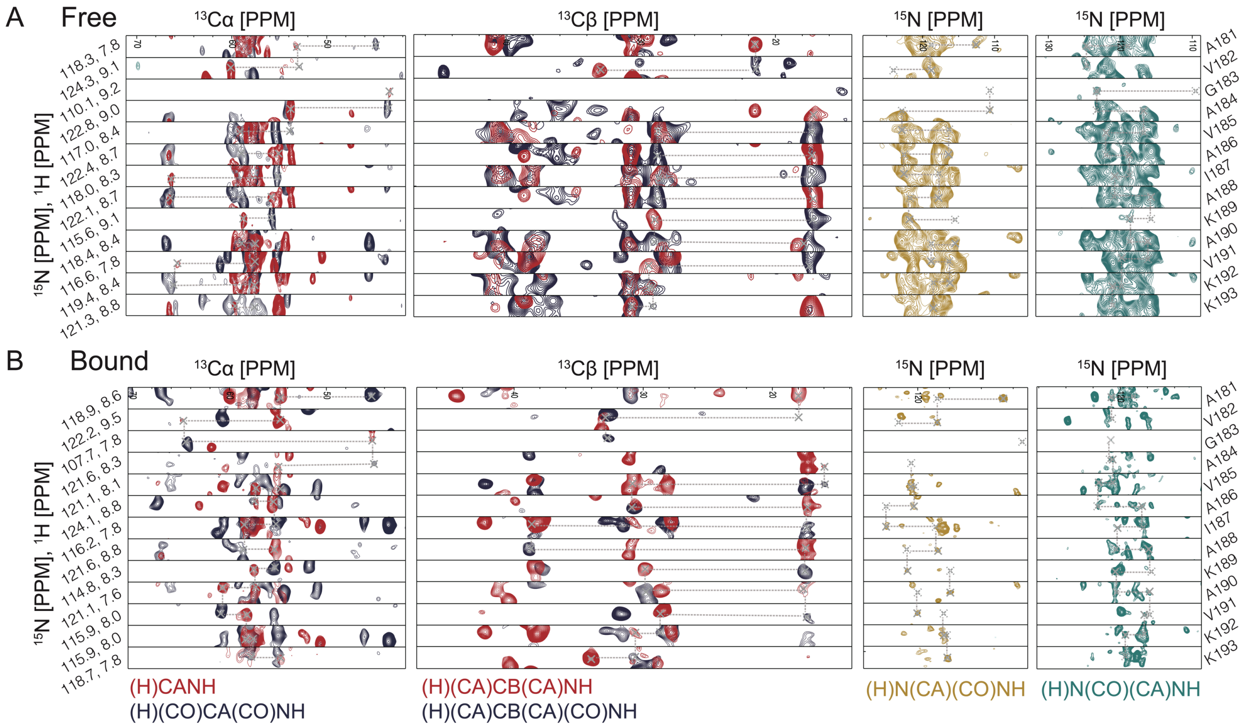

Supplementary Figure 4. Sequence specific assignment of CitApc residues A181 to K193 in citrate free (A) and citrate bound (B) state. The assignment process using (H)CANH, (H)(CO)CA(C))NH, (H)(CA)CB(CA)NH, (H)(CA)CB(CA)(CO)NH, (H)N(CA)(CO)NH and (H)N(CO)(CA)NH is shown. The same manual assignment process was done throughout CitApc in both states. The Cβ chemical shifts of K192 and K193 in the (H)(CA)CB(CA)NH spectra change to higher values with citrate binding. This indicates that the K192 and K193 residues have higher helix forming propensity in the citrate free state.

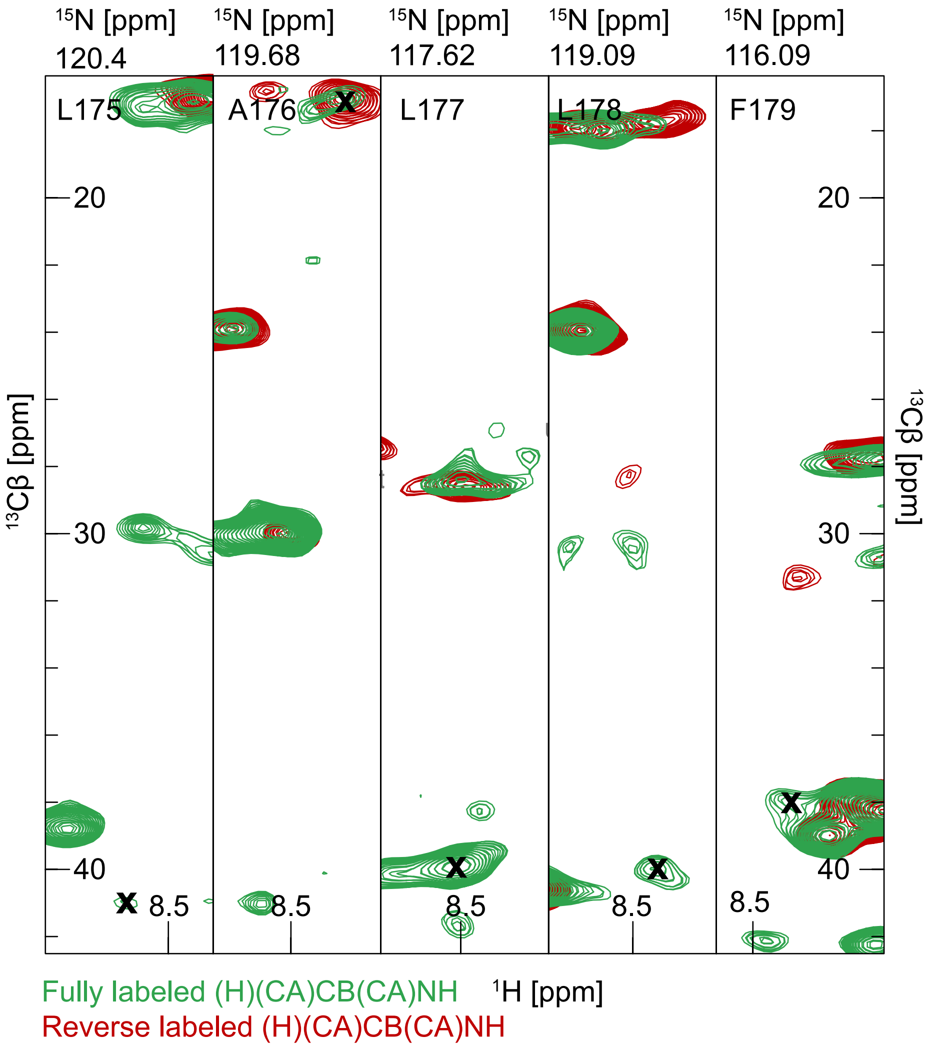

Supplementary Figure 5. The IVFL reverse labeled CitApc sample helps with residue typing in the manual assignment process in particular in the TM helices. An example is shown for the comparison of H(CA)CB(CA)NH spectra in both fully labeled (green) and reverse labeled (red) for residue L175 to F179 in the citrate bound state (peak position marked with bold x in each strip). The change in peak intensity along with the characteristic chemical shifts untangles the ambiguity of the assignment as the signal intensity for L175, L177, F179 disappeared, while the non-reverse labeled residue A176 remains the same.

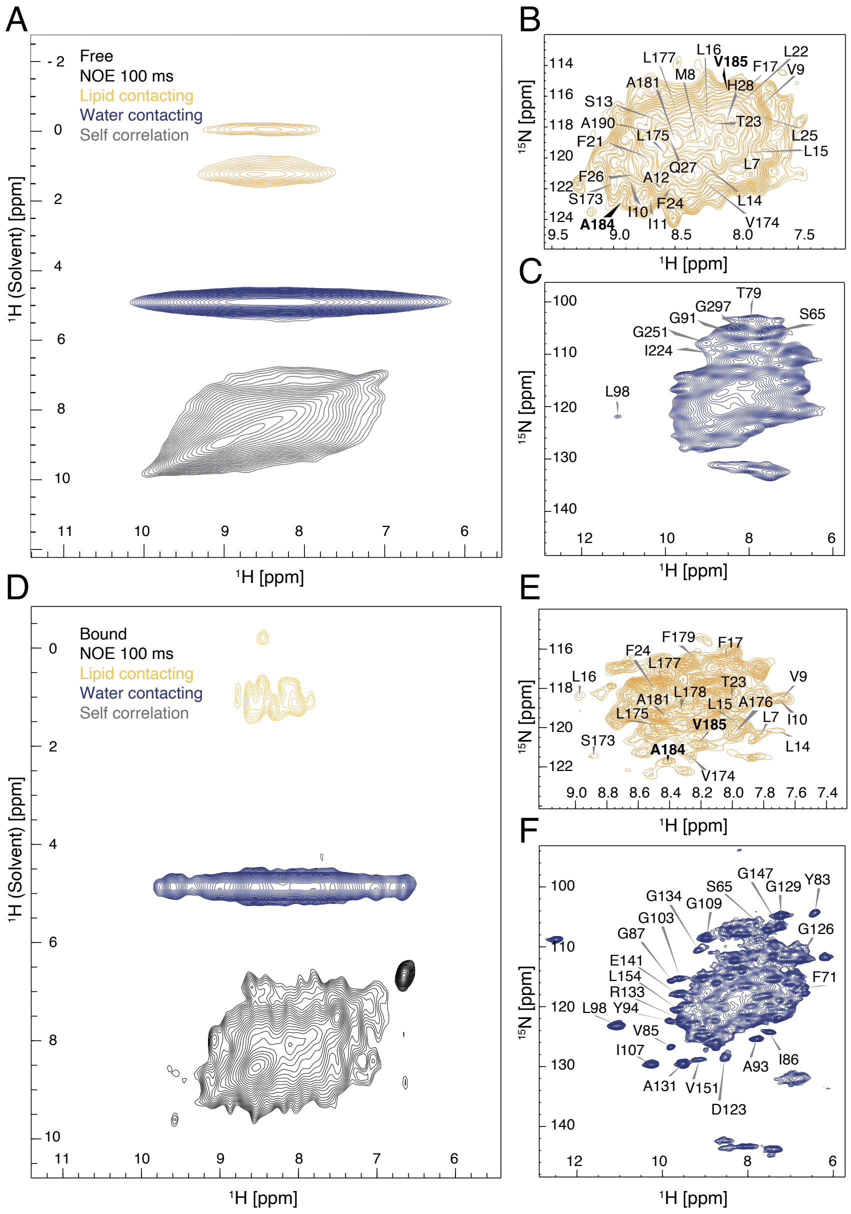

Supplementary Figure 6. (H)HNH lipid contacting spectra of CitApc confirming chemical shift assignment of the TM helices in the citrate free (A, B, C) and citrate bound (D, E, F) state. The additional proton dimension separates the signal from the proton and amide nitrogen dimension into water contacting (blue) and lipid contacting (yellow) parts (A, D). The protein-protein 'self-correlation' signals are shown in grey. The lipid tail group (0.7 ppm) contacting region in the free state has more intensity than in the bound state. The assigned TM helical resonances can be checked if they are contacting lipid on the H-N planes (B, E). The water (4.7 ppm) contacting region (C, F) in each state appears the same as the corresponding (H)NH spectrum since isolated peaks mostly come from either PASp or PASc, both of which have substantial exposure to water.

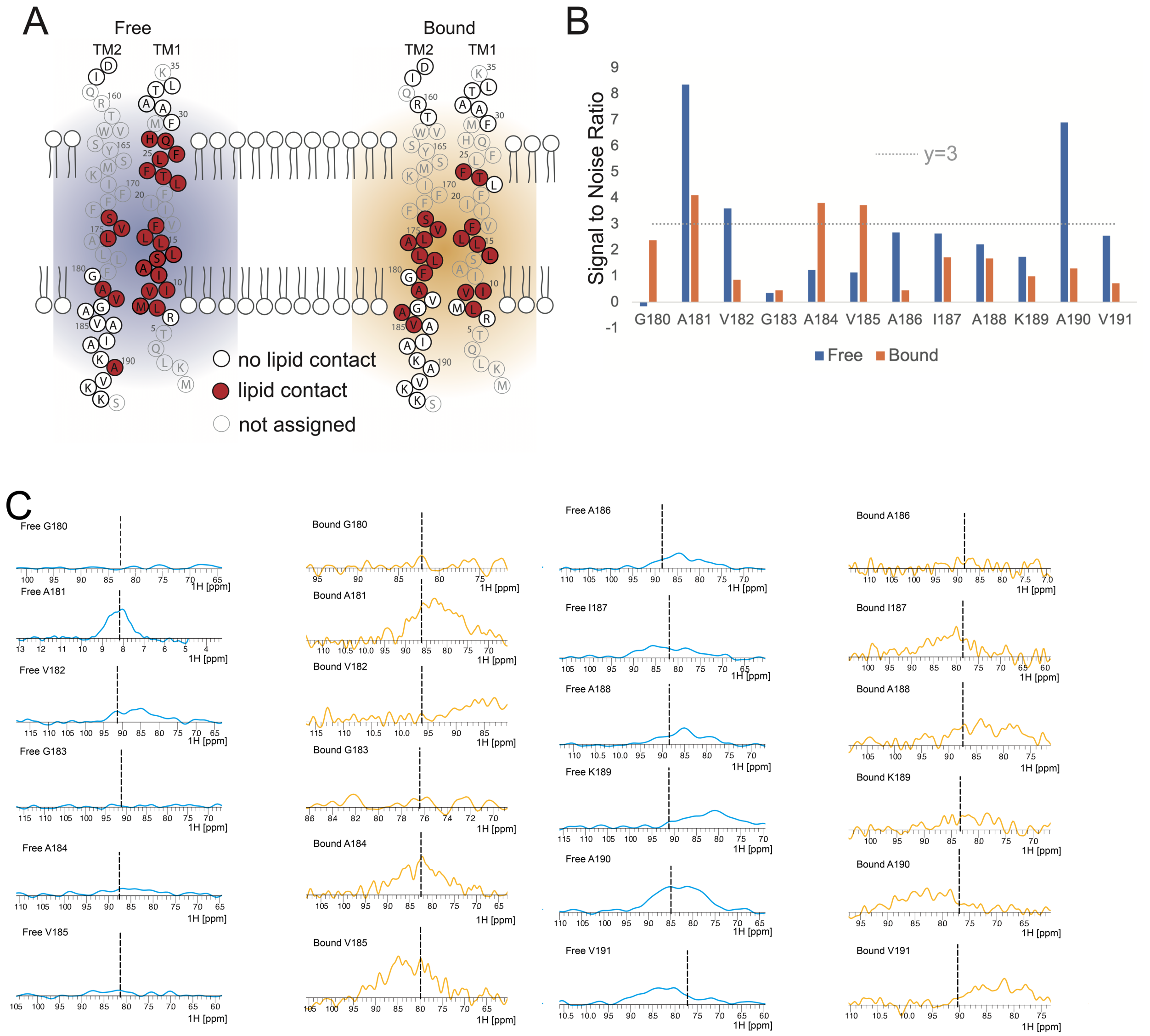

Supplementary Figure 7. Differential lipid contacts of CitA TM residues. (A) Lipid contacting residues in the TM helices mapped onto the CitA topology map in the free (left, blue) and bound (right, yellow) state of CitA. (B) Peak signal to noise ratio of residues G180 to V191 in the (H)HNH lipid contacting spectra in the free (blue) and bound (yellow) state. Residues A184 and V185 show lipid contacts in the bound state but not in the free state. Residue A190 appears to lose a contact upon citrate binding, however, this change might also be explained by differences in overlap with other peaks in the spectrum (Supplementary Figure 6 B and E). Peaks with signal to noise ratio larger than 3 (dashed grey line) are considered lipid contacting. The border of lipid contact in the TM1 helix does not change while the border of lipid contact in the TM2 helix is shifted consistent with additional c-terminal residues entering the membrane. (C) Slices taken from Supplementary Figure 5 B and E, showing the peak positions used for the analysis in panel B . Note the spectral crowding compromises quantitation of several cross-peaks, but that residues 184-185 are resolved in Supplementary Figure 6E.

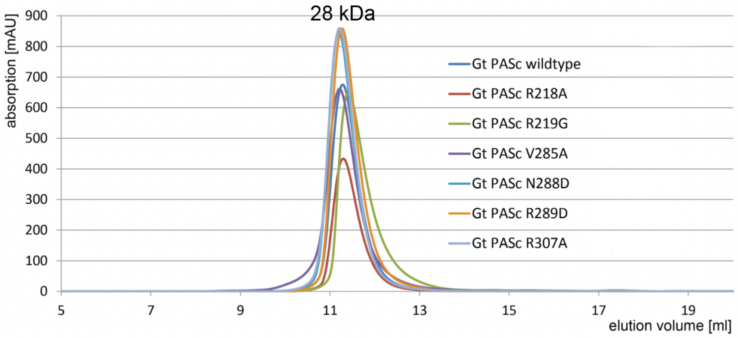

Supplementary Figure 8. Size exclusion chromatography profile of G.thermodenitrificans CitA PASc mutants.

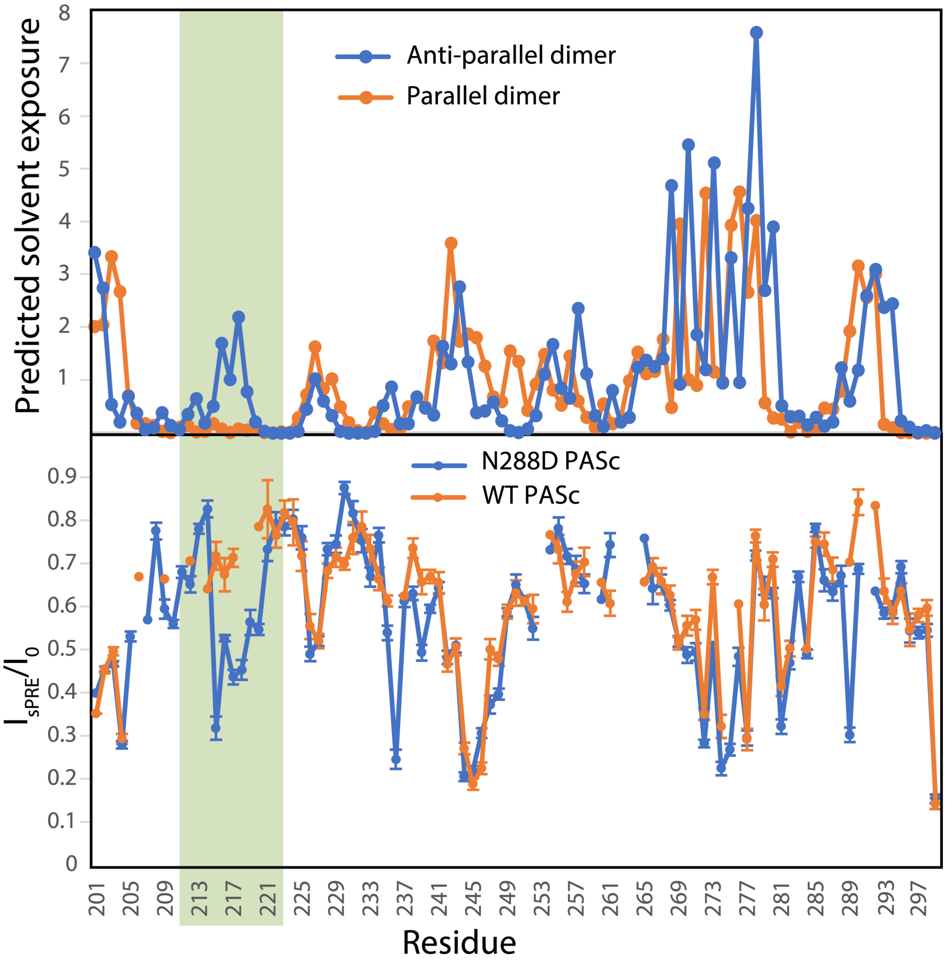

Supplementary Figure 9. Predicted solvent exposure of the anti-parallel and parallel dimers from the crystals and the measured intensity ratios of the N288D mutant and WT PASc in solution. (Top) Predicted solvent exposure of the anti-parallel dimer (blue) and parallel dimer (orange) from crystal structures of the N288D mutant and WT PASc. (Bottom) 15N-HSQC peak intensity ratios between the paramagnetic (IsPRE) and diamagnetic (I0) samples of the N288D mutant (blue) and WT (orange) PASc in solution. The sPRE profiles of two dimer forms are different from each other mainly at the N-terminal helix (green box). In this region, the anti-parallel dimer (top panel, blue curve) is predicted to have significantly higher solvent exposure, which corresponds to the lower intensity ratios in the N288D mutant PASc N-terminal helix in solution (bottom panel, blue curve).

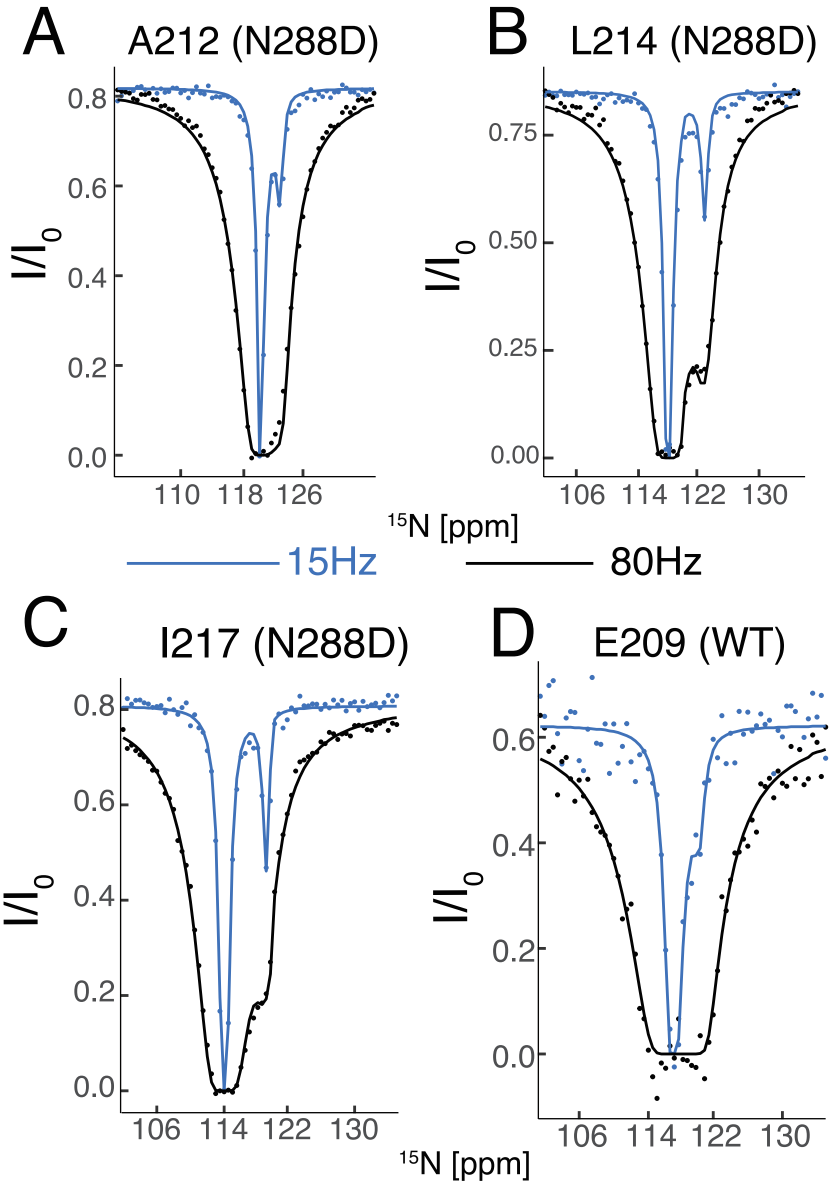

Supplementary Figure 10. CEST profiles and fitting of residues (A) A212 in N288D PASc, (B) L214 in N288D PASc, (C) I217 in N288D PASc and (D) E209 in WT PASc.

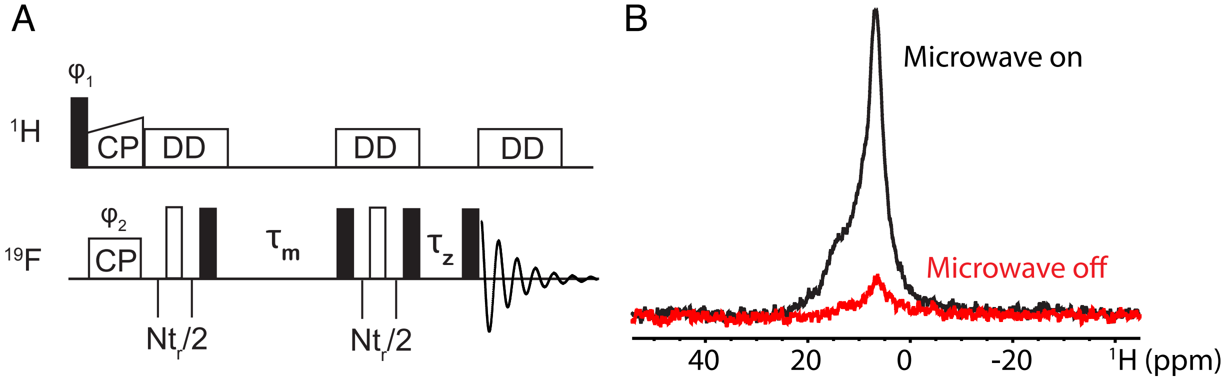

Supplementary Figure 11. DNP enhanced CODEX measurement. A) Pulse sequence for the HF CODEX measurement. T_1 compensation (τ_Z) and mixing time (τ_m) was set according to previous literature [58,59]. A cross polarization (CP) contact time of 1.5 ms was used. For a proper recoupling of ^{19}F chemical shift anisotropy (CSA), N in the pulse sequence was set to 7. The sample temperature was 90 K, and the instrument a 600 MHz Bruker widebore spectrometer. The probe was a 2.5 mm Phoenix NMR (Loveland, CO) HFXY MAS DNP probe with MAS set to 20 kHz. B) a proton spectrum was used to characterized the signal enhancement from DNP. 8-fold enhancement was observed.

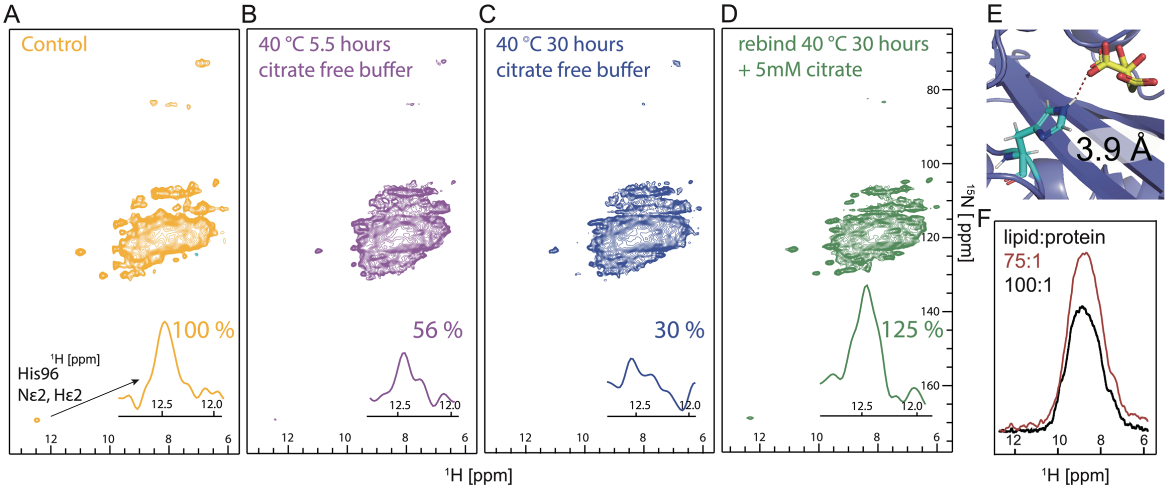

Supplementary Figure 12. Citrate is removed from CitApc through incubation in citrate free buffer as tracked by the peak intensity of the H96 side chain. (A) The citrate-bound spectrum. (B to C) the intensity drops to 30 % after 30 hours of incubation. (D) Addition of citrate to the buffer immediately restores the spectra to the bound state, indicating the reversibility of the protocol. (F) This citrate removal protocol optimizes sensitivity of the citrate free state, as it (red) increases the sensitivity by 30 % compared with the original protocol (black), which used a higher lipid to protein ratio of 100:1. (E) the hydrogen bonding interaction between H96 and citrate is shown as a dashed line. The loss of this hydrogen bond causes the H96 side chain to become unstructured and thus too dynamic to be detected by CP based (H)NH experiment.

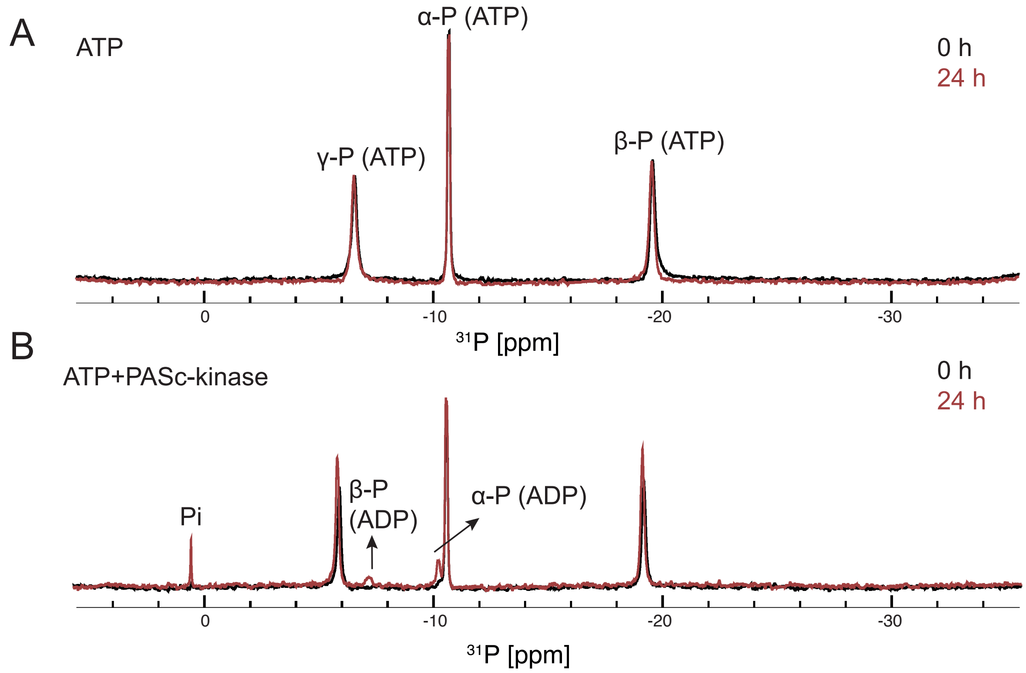

Supplementary Figure 13. The addition of the PASc-kin WT construct hydrolyzes ATP into ADP (B), which is otherwise stable under the experimental conditions (A). The 1D 31P spectrum of ATP does not change after 24 hours, indicating the stability of the ATP under the experimental conditions. The spectrum shows three peaks, corresponding to the signals of β-P, α-P and γ-P. The addition of PASc-kinase leads to the appearance of signals of the degradation products ADP (β-P and α-P in ADP) and free phosphate (Pi).

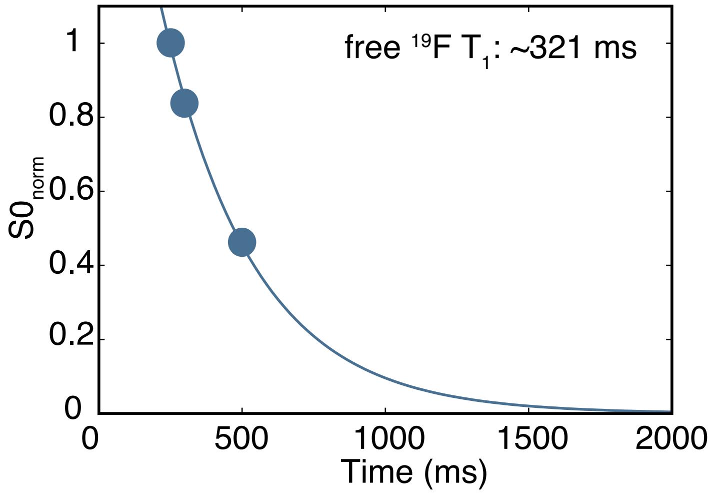

Supplementary Figure 14. The T_1 of ^{19}F is 321 ms in the citrate free state, explaining the very weak signal observed with more than 500 ms of CODEX.

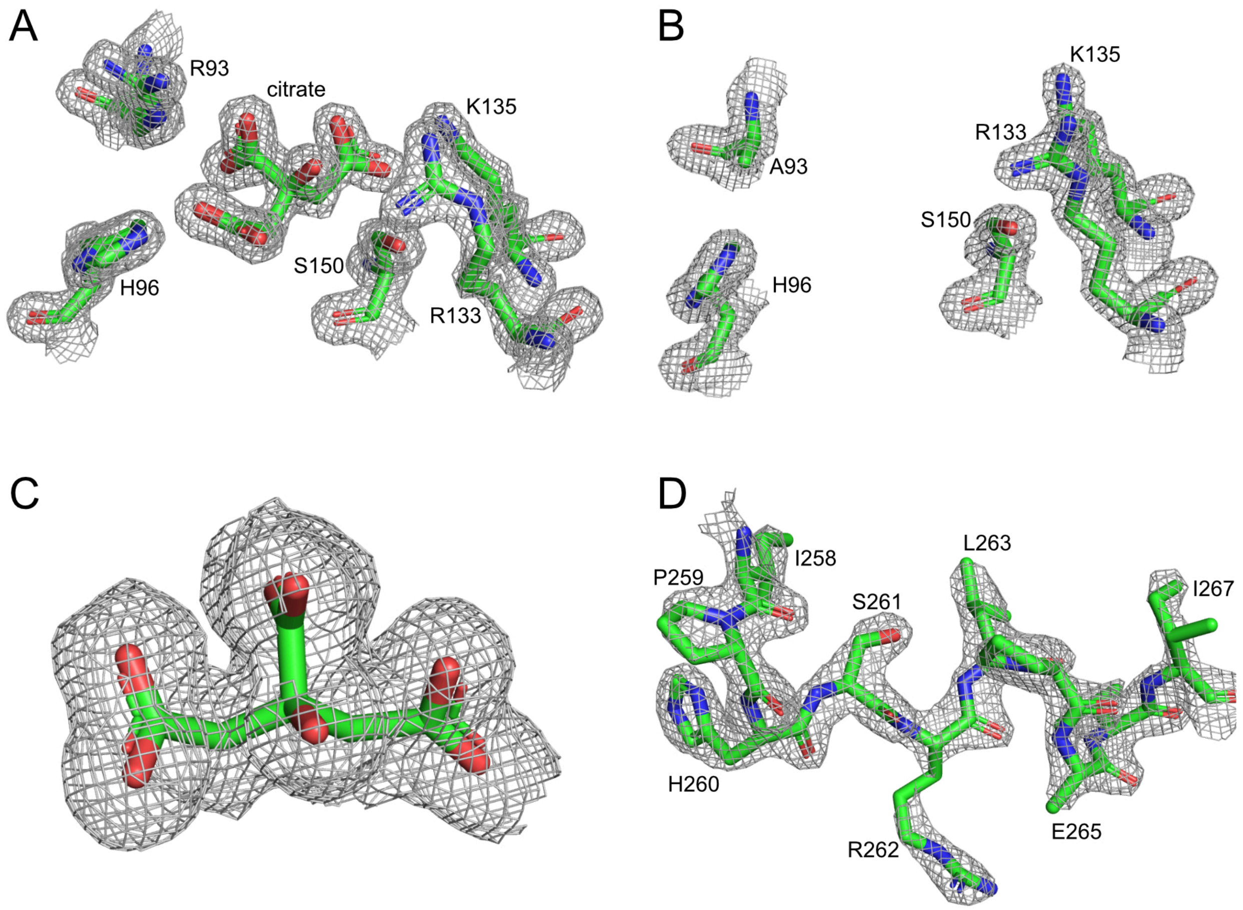

Supplementary Figure 15. Representative electron densities of the crystal structures solved in this work. (A, B) Residues interacting with citrate in the Gt PASp structure (A) and in the Gt PASp R93A mutant structure, where citrate is not bound. (C) OMIT map of citrate in the Gt PASp structure. (D) Electron density in a loop region of the Gt PASc N288D mutant structure. The maps in A, B and D are $2F_o$-Fc maps. All maps are contoured at 2.0 sigma, within 1.6 Å of the selected atoms.

Supplementary Table 1. Primers for Gt CitA construct design and mutagenesis

Primer	Nucleotide sequence 5′ → 3′ (restriction sites in bold)
CitA N-Terminus	GCT TCT AGA **CAT ATG** AAG CTT CAG ACA AGG TTA ATG GTC
CitA C-Terminus	GTC GAA TTC **GGA TCC** TCA ATG GTG ATT GGC GTC GGC
CitA C12A forward	CAG ACA AGG TTA ATG GTC ATT ATT GCT TCC CTG CTA TTA TTT GTG ATC
CitA C12A reverse	GAT CAC AAA TAA TAG CAG GGA AGC AAT AAT GAC CAT TAA CCT TGT CTG
CitA R93A forward	CCG ACA AGG CAT TGC TTA TGC CCA TCC GC
CitA R93A reverse	GCG GAT GGG CAT AAG CAA TGC CTT GTC GG
CitA N308C forward	GCG GTA TCG ACG TTT CGC TGC AAG TCG GAG TTG TAC CGC
CitA N308C reverse	GCG GTA CAA CTC CGA CTT GCA GCG AAA CGT CGA TAC CGC
CitA N288D forward	GAG GAG AGA CCG TCA TTG CGG ACA GGA TTC CGA TCA AAA AC
CitA N288D reverse	GTT TTT GAT CGG AAT CCT GTC CGC AAT GAC GGT CTC TCC TC

Supplementary Table 2. NMR experimental parameters used in sequence specific assignment in the citrate free state of CitApc. The powers for soft pulses are taken at the maximum value.

Spectrum	(H)NH	(H)CANH	(H)(CO)CA(CO)NH	(H)CONH	(H)CO(CA)NH
Scans per point	8	24	96	8	96
Experimental time / h	0.9	112	205.3	17.2	206.4
Signal/noise	85.99	28.22	10.29	20.85	15.06
Field	850	850	850	850	850
Spinning frequency/Hz	55000	55000	55000	55000	55000
interscan delay /s	1	1	1	1	1
Sweep width (t1) / ppm	160 (N)	32 (N)	40 (N)	40 (N)	40 (N)
Max indirect evolution (t1) / ms	14.5 (N)	21.8 (N)	10.1 (N)	12.5 (N)	12.5 (N)
Sweep width (t2) / ppm	40.3 (H)	32 (C)	30 (C)	18.0 (C)	18.0 (C)
Max indirect evolution (t2) / ms	30.0 (H)	10.2 (C)	8.6 (C)	11.7 (C)	11.7 (C)
Sweep width (t3) / ppm	N/A	58.8 (H)	40.3 (H)	40.3 (H)	40.3 (H)
Max indirect evolution (t3) / ms	N/A	20.5 (H)	30.0 (H)	30.0 (H)	30.0 (H)
**Transfer I**	**HN (dipolar)**	**HCA (dipolar)**	**HCO (dipolar)**	**HCO (dipolar)**	**HCO (dipolar)**
^{1}H field / kHz	99.4	95.1	96.3	100	100
X field / kHz	38.1	40.4	40.1	40.1	40.1
Shape	100 - 80 % ramp (H)	85 - 100 % ramp (H)	85 - 100 % ramp (H)	85 - 100 % ramp (H)	85 - 100 % ramp (H)
Carrier ^{13}C	N/A	53.7	173.3	173.8	173.8
Time / ms	1.15	6	3.5	3.5	3.5
**Transfer II**	**HN (dipolar)**	**CAN (dipolar)**	**COCA (scalar)**	**CON (dipolar)**	**COCA (scalar, SCT)**
^{1}H field / kHz	95.5	N/A	N/A	N/A	N/A
^{13}C field / kHz	N/A	35.5	N/A	27.4	N/A
^{15}N field / kHz	38.1	29.2	N/A	31.6	N/A

Spectrum	(H)NH	(H)CANH	(H)(CO)CA(CO)NH	(H)CONH	(H)CO(CA)NH
Carrier ^{13}C	N/A	N/A	173.3 and 53.3	173.8	173.8 and 53.3
Shape	100 - 80 % ramp (H)	Tan_ampmod_63_94.land (N)		Tan_ampmod_63_94.land (N)	
Time / ms	0.85	11	13.2	9	6.6 (1st step)<br>4.6 (2nd step)
**Transfer III**		**NH (dipolar)**	**CON (dipolar)**	**NH (dipolar)**	**CAN (dipolar)**
^{1}H field / kHz	N/A	98.1	N/A	98.1	N/A
^{13}C field / kHz	N/A	N/A	27.4	N/A	27.4
^{15}N field / kHz	N/A	38.6	30.3	38.1	30.3
Carrier ^{13}C	N/A	N/A	173.3	N/A	53.3
Shape	N/A	100 - 80 % ramp (H)	Tan_ampmod_63_94.land (N)	100 - 80 % ramp (H)	Tan_ampmod_63_94.land (N)
Time / ms	N/A	0.85	9	0.85	9
**Transfer IV**			**NH (dipolar)**		**NH (dipolar)**
^{1}H field / kHz	N/A	N/A	93.7	N/A	98.1
^{13}C field / kHz	N/A	N/A	N/A	N/A	N/A
^{15}N field / kHz	N/A	N/A	38.1	N/A	38.1
Carrier ^{13}C	N/A	N/A	N/A	N/A	N/A
Shape	N/A	N/A	100 - 80 % ramp (H)	N/A	100 - 80 % ramp (H)
Time / ms	N/A	N/A	0.85	N/A	0.85
interscan delay /s	1	1	1	1	1

Spectrum	(H)(CA)CB(CA)NH	(H)(CA)CB(CA)(CO)NH	(H)N(CA)(CO)NH
Scans per point	12	88	96
Experimental time / h	102	322.7	116
Signal/noise	N/A	N/A	6.91
Field	850	850	850
Spinning frequency/Hz	55000	55000	55000
interscan delay /s	1	1	1
Sweep width (t1) / ppm	32(N)	40(N)	40.0 (N)
Max indirect evolution (t1) / ms	16.3 (N)	9.6 (N)	9.6 (N)
Sweep width (t2) / ppm	90.0 (C)	84 (C)	40.0 (N)

Spectrum	(H)(CA)CB(CA)NH	(H)(CA)CB(CA)(CO)NH	(H)N(CA)(CO)NH
Max indirect evolution (t2) / ms	8.8 (C)	5.6 (C)	9.6 (N)
Sweep width (t3) / ppm	58.8 (H)	40.3 (H)	40.3 (H)
Max indirect evolution (t3) / ms	20.5 (H)	30.0 (H)	30.0 (H)
**Transfer I**	**HCA (dipolar)**	**HCA (dipolar)**	**HN (dipolar)**
^{1}H field / kHz	95.1	98.1	100
X field / kHz	40.4	40.1	38.7
Shape	85 - 100 % ramp (H)	85 - 100 % ramp (H)	100 - 80 % ramp (H)
Carrier ^{13}C	53.7	53.7	N/A
Time / ms	7	3.5	1.15
**Transfer II**	**CACBCA(scalar)**	**CACBCA(scalar)**	**NCA (dipolar)**
^{1}H field / kHz	N/A	N/A	N/A
^{13}C field / kHz	N/A	N/A	27.4
^{15}N field / kHz	N/A	N/A	29.4
Carrier ^{13}C	53.7 and 48	53.7 and 48	53.3
Shape	N/A	N/A	Tan_ampmod_63_94.land (N)
Time / ms	12	12	9
**Transfer III**	**CAN (dipolar)**	**COCA (scalar)**	**CACO (scalar)**
^{1}H field / kHz	N/A	N/A	N/A
^{13}C field / kHz	35.5	N/A	N/A
^{15}N field / kHz	29.2	N/A	N/A
Carrier ^{13}C	53.3	173.3 and 53.3	53.3 and 173.3
Shape	Tan_ampmod_63_94.land (N)		N/A
Time / ms	11	6.6 (1st step) 4.6 (2nd step)	4.6 (1st step) 6.6 (2nd step)
**Transfer IV**	**NH (dipolar)**	**CON (dipolar)**	**CON (dipolar)**
^{1}H field / kHz	98.1	N/A	N/A
^{13}C field / kHz	N/A	27.4	27.4
^{15}N field / kHz	38.6	28.9	30.1
Carrier ^{13}C	N/A	173.3	173.3

Spectrum	(H)(CA)CB(CA)NH	(H)(CA)CB(CA)(CO)NH	(H)N(CA)(CO)NH
Shape	100 - 80 % ramp (H)	Tan_ampmod_63_94.I and (N)	Tan_ampmod_63_94.land (N)
Time / ms	0.85	9	9
**Transfer V**		**NH (dipolar)**	**NH (dipolar)**
^{1}H field / kHz	N/A	100	95.1
^{13}C field / kHz	N/A	N/A	N/A
^{15}N field / kHz	N/A	38.1	38.7
Carrier ^{13}C	N/A	N/A	N/A
Shape	N/A	100 - 80 % ramp (H)	100 - 80 % ramp (H)
Time / ms	N/A	0.85	0.85

Spectrum	(H)N(CO)(CA)NH	(H)COCA(N)H	H(H)NH
Scans per point	128	72	8
Experimental time / h	154.9	158.1	22
Signal/noise	7.85	11.9	104.84
Field	850	850	850
Spinning frequency/Hz	55000	55000	55000
interscan delay /s	1	1	1
Sweep width (t1) / ppm	40.0 (N)	18.0 (CO)	50.0 (N)
Max indirect evolution (t1) / ms	9.6 (N)	9.9 (CO)	12.3 (N)
Sweep width (t2) / ppm	40.0 (N)	30 (CA)	15.0 (H)
Max indirect evolution (t2) / ms	9.6 (N)	8.1 (CA)	3.5 (H)
Sweep width (t3) / ppm	40.3 (H)	40.3 (H)	40.3 (H)
Max indirect evolution (t3) / ms	30.0 (H)	30.0 (H)	30.0 (H)
**Transfer I**	**HN (dipolar)**	**HCO (dipolar)**	**HH (NOE)**
^{1}H field / kHz	100	96.1	N/A
X field / kHz	38.7	40.1	N/A
Shape	100 - 80 % ramp (H)	85 - 100 % ramp (H)	N/A
Carrier ^{13}C	N/A	173.3	N/A
Time / ms	1.15	3.5	100

Spectrum	(H)N(CO)(CA)NH	(H)COCA(N)H	H(H)NH
**Transfer II**	**NCO(dipolar)**	**COCA (scalar, SCT)**	**HN (dipolar)**
^{1}H field / kHz	N/A	N/A	98.4
^{13}C field / kHz	27.4	N/A	N/A
^{15}N field / kHz	30.1	N/A	40.5
Carrier ^{13}C	173.3	173.3 then 53.3	N/A
Shape	Tan_ampmod_63_94.land (N)		80 - 100 % ramp (H)
Time / ms	9	6.6 (1st step) 4.6 (2nd step)	**1.15**
**Transfer III**	**COCA (scalar)**	**CAN (dipolar)**	**HN (dipolar)**
^{1}H field / kHz	N/A	N/A	95
^{13}C field / kHz	N/A	27.4	N/A
^{15}N field / kHz	N/A	27.8	40.5
Carrier ^{13}C	173.3 and 53.3	53.3	N/A
Shape	N/A	Tan_ampmod_63_94.land (N)	100 - 80 % ramp (H)
Time / ms	6.6 (1st step) 4.6 (2nd step)	9	0.85
**Transfer IV**	**CAN (dipolar)**	**NH (dipolar)**	
^{1}H field / kHz	N/A	101.9	N/A
^{13}C field / kHz	27.4	N/A	N/A
^{15}N field / kHz	29.4	38.1	N/A
Carrier ^{13}C	53.3	N/A	N/A
Shape	Tan_ampmod_63_94.land (N)	100 - 80 % ramp (H)	N/A
Time / ms	9	0.85	N/A
**Transfer V**	**NH (dipolar)**		
^{1}H field / kHz	95.1	N/A	N/A
^{13}C field / kHz	N/A	N/A	N/A
^{15}N field / kHz	38.7	N/A	N/A
Carrier ^{13}C	N/A	N/A	N/A
Shape	100 - 80 % ramp (H)	N/A	N/A
Time / ms	0.85	N/A	N/A

Supplementary Table 3. NMR experimental parameters used in sequence specific assignment in the citrate bound state of CitApc. The powers for soft pulses are taken at the maximum value.

Spectrum	(H)NH	(H)CANH	(H)(CO)CA(CO)NH	(H)CONH	(H)CO(CA)NH
Scans per point	64	8	56	24	64
Experimental time / h	15.9	57.6	131.4	61.9	144.9
Signal/noise	232	20.64	12.65	22.99	8.57
Field	850	850	850	850	850
Spinning frequency/Hz	55000	55000	55000	55000	55000
interscan delay /s	3.5	2.5	1	1	0.74
Sweep width (t1) / ppm	120.0 (N)	36 (N)	36 (N)	34(N)	34(N)
Max indirect evolution (t1) / ms	24.8 (N)	17.4 (N)	15.5 (N)	18.4 (N)	18.4 (N)
Sweep width (t2) / ppm	40.8 (H)	32 (C)	32 (C)	16.0 (C)	16.0 (C)
Max indirect evolution (t2) / ms	29.5 (H)	7.0 (C)	6.4 (C)	12.6 (C)	12.6 (C)
Sweep width (t3) / ppm	N/A	58.8 (H)	58.8 (H)	58.8 (H)	58.8 (H)
Max indirect evolution (t3) / ms	N/A	20.5 (H)	20.5 (H)	20.5 (H)	20.5 (H)
**Transfer I**	**HN (dipolar)**	**HCA (dipolar)**	**HCO (dipolar)**	**HCO (dipolar)**	**HCO (dipolar)**
^{1}H field / kHz	96.4	94.35	93.1	91.8	94.1
X field / kHz	70.7	40	40	39.8	40.1
Shape	85 - 100 % ramp (H)	85 - 100 % ramp (H)	85 - 100 % ramp (H)	85 - 100 % ramp (H)	85 - 100 % ramp (H)
Carrier ^{13}C		53.7	173.3	173.8	173.8
Time / ms	1.2	4	5	4	4.2
**Transfer II**	**HN (dipolar)**	**CAN (dipolar)**	**COCA (scalar)**	**CON (dipolar)**	**COCA (scalar)**
^{1}H field / kHz	96.4	N/A	N/A	N/A	N/A
^{13}C field / kHz	N/A	34.35	N/A	34.4	N/A
^{15}N field / kHz	70.7	30.61	N/A	31.5	N/A
Carrier ^{13}C	N/A	53.7	173.3 and 53.3	173.8	173.8 and 53.3

Spectrum	(H)NH	(H)CANH	(H)(CO)CA(CO)NH	(H)CONH	(H)CO(CA)NH
Shape	80 - 100 % ramp (H)	Tan_ampmod _63_94.land (N)	N/A	Tan_ampmod_ 63_94.land (N)	N/A
Time / ms	1.2	13	15	13	7.46
**Transfer III**		**NH (dipolar)**	**CON (dipolar)**	**NH (dipolar)**	**CAN (dipolar)**
^{1}H field / kHz	N/A	96.45	N/A	95.1	N/A
^{13}C field / kHz	N/A	N/A	34.6	N/A	34.9
^{15}N field / kHz	N/A	39.41	32	38.7	30.1
Carrier ^{13}C	N/A	N/A	173.3	N/A	53.3
Shape	N/A	100 - 80 % ramp (H)	Tan_ampmod_63_ 94.land (N)	100 - 80 % ramp (H)	Tan_ampmod_ 63_94.land (N)
Time / ms	N/A	1.2	11.5	0.85	13
**Transfer IV**			**NH (dipolar)**		**NH (dipolar)**
^{1}H field / kHz	N/A	N/A	96.45	N/A	99.2
^{13}C field / kHz	N/A	N/A	N/A	N/A	N/A
^{15}N field / kHz	N/A	N/A	39.41	N/A	38.7
Carrier ^{13}C	N/A	N/A	N/A	N/A	N/A
Shape	N/A	N/A	100 - 80 % ramp (H)	N/A	100 - 80 % ramp (H)
Time / ms	N/A	N/A	1.15	N/A	0.7

Spectrum	(H)(CA)CB(CA)NH	(H)(CA)CB(CA)(CO)NH	(H)N(CA)(CO)NH
Scans per point	48	48	88
Experimental time / h	296.3	269.3	262.9
Signal/noise	N/A	N/A	14.81
Field	850	850	800
Spinning frequency/Hz	55000	55000	55000
interscan delay /s	1	1	1
Sweep width (t1) / ppm	34(N)	34(N)	36.0 (N)
Max indirect evolution (t1) / ms	18.4 (N)	17.4 (N)	16.5 (N)
Sweep width (t2) / ppm	70.0 (C)	70.0 (C)	36.0 (N)
Max indirect evolution (t2) / ms	6.7 (C)	6.6 (C)	16.1 (N)

Spectrum	(H)(CA)CB(CA)NH	(H)(CA)CB(CA)(CO)NH	(H)N(CA)(CO)NH
Sweep width (t3) / ppm	58.8 (H)	58.8 (H)	30.1 (H)
Max indirect evolution (t3) / ms	20.5 (H)	20.5 (H)	21.3 (H)
**Transfer I**	**HCA (dipolar)**	**HCA (dipolar)**	**HN (dipolar)**
^{1}H field / kHz	94.7	94.4	104.2
X field / kHz	40	38.7	39.1
Shape	85 - 100 % ramp (H)	85 - 100 % ramp (H)	80 - 100 % ramp (H)
Carrier ^{13}C	53.7	53.7	N/A
Time / ms	4	4	1
**Transfer II**	**CACBCA(scalar)**	**CACBCA(scalar)**	**NCA (dipolar)**
^{1}H field / kHz	N/A	N/A	N/A
^{13}C field / kHz	N/A	N/A	20.9
^{15}N field / kHz	N/A	N/A	43.7
Carrier ^{13}C	53.7 and 48	53.7 and 48	53.3
Shape	N/A	N/A	Tan_ampmod_63_94.land (N)
Time / ms	18.56	18.56	13
**Transfer III**	**CAN (dipolar)**	**COCA (scalar)**	**CACO (scalar)**
^{1}H field / kHz	N/A	N/A	N/A
^{13}C field / kHz	34.8	N/A	N/A
^{15}N field / kHz	30.6	N/A	N/A
Carrier ^{13}C	53.7	173.3 and 53.3	53.3 and 173.3
Shape	Tan_ampmod_63_94.land (N)		N/A
Time / ms	13	7.2 (1st step) 6.4 (2nd step)	6.4 (1st step) 7.2 (2nd step)
**Transfer IV**	**NH (dipolar)**	**CON (dipolar)**	**CON (dipolar)**
^{1}H field / kHz	95.4	N/A	N/A
^{13}C field / kHz	N/A	34.4	18.4
^{15}N field / kHz	39.4	30.6	46.5
Carrier ^{13}C	N/A	173.3	173.3
Shape	100 - 80 % ramp (H)	Tan_ampmod_63_94.land (N)	Tan_ampmod_63_94.land (N)

Spectrum	(H)(CA)CB(CA)NH	(H)(CA)CB(CA)(CO)NH	(H)N(CA)(CO)NH
Time / ms	1.15	13	13
**Transfer V**		**NH (dipolar)**	**NH (dipolar)**
^{1}H field / kHz	N/A	96.4	95.6
^{13}C field / kHz	N/A	N/A	N/A
^{15}N field / kHz	N/A	39.4	39.1
Carrier ^{13}C	N/A	N/A	N/A
Shape	N/A	100 - 80 % ramp (H)	100 - 80 % ramp (H)
Time / ms	N/A	1.15	0.6

Spectrum	(H)N(CO)(CA)NH	(H)COCA(N)H	H(H)NH
Scans per point	144	64	16
Experimental time / h	338.6	159	102.4
Signal/noise	11.19	13.53	66.4
Field	850	850	850
Spinning frequency/Hz	55000	55000	55000
interscan delay /s	1	1	1
Sweep width (t1) / ppm	36.0 (N)	16.0 (CO)	60.0 (N)
Max indirect evolution (t1) / ms	14.8 (N)	12.3 (CO)	24.8 (N)
Sweep width (t2) / ppm	36.0 (N)	32 (CA)	15.0 (H)
Max indirect evolution (t2) / ms	14.8 (N)	7.6 (CA)	3.5 (H)
Sweep width (t3) / ppm	58.8 (H)	58.8 (H)	40.8 (H)
Max indirect evolution (t3) / ms	20.5 (H)	20.5 (H)	29.5 (H)
**Transfer I**	**HN (dipolar)**	**HCO (dipolar)**	**HH (NOE)**
^{1}H field / kHz	96	91.5	N/A
X field / kHz	38.7	39.8	N/A
Shape	100 - 80 % ramp (H)	85 - 100 % ramp (H)	N/A
Carrier ^{13}C	N/A	173.3	N/A
Time / ms	1.15	4	100
**Transfer II**	**NCO(dipolar)**	**COCA (scalar, SCT)**	**HN (dipolar)**

Spectrum	(H)N(CO)(CA)NH	(H)COCA(N)H	H(H)NH
^{1}H field / kHz	N/A	N/A	93.6
^{13}C field / kHz	27.1	N/A	N/A
^{15}N field / kHz	37.7	N/A	38.7
Carrier ^{13}C	173.3	173.3 then 53.3	N/A
Shape	Tan_ampmod_63_94.land (N)		100 - 80 % ramp (H)
Time / ms	13	7.2 (1st step) 6.4 (2nd step)	0.7
**Transfer III**	**COCA (scalar)**	**CAN (dipolar)**	**HN (dipolar)**
^{1}H field / kHz	N/A	N/A	94.2
^{13}C field / kHz	N/A	35	N/A
^{15}N field / kHz	N/A	29.7	38.7
Carrier ^{13}C	173.3 and 53.3	N/A	N/A
Shape	N/A	Tan_ampmod_63_94.land (N)	100 - 80 % ramp (H)
Time / ms	7.2 (1st step) 6.4 (2nd step)	12.5	0.6
**Transfer IV**	**CAN (dipolar)**	**NH (dipolar)**	
^{1}H field / kHz	N/A	95.1	N/A
^{13}C field / kHz	35.1	N/A	N/A
^{15}N field / kHz	30.1	38.7	N/A
Carrier ^{13}C	53.3	N/A	N/A
Shape	Tan_ampmod_63_94.land (N)	100 - 80 % ramp (H)	N/A
Time / ms	12.5	0.85	N/A
**Transfer V**	**NH (dipolar)**		
^{1}H field / kHz	95.1	N/A	N/A
^{13}C field / kHz	N/A	N/A	N/A
^{15}N field / kHz	38.7	N/A	N/A
Carrier ^{13}C	N/A	N/A	N/A
Shape	100 - 80 % ramp (H)	N/A	N/A
Time / ms	0.85	N/A	N/A

Supplementary Table 4. NMR experimental parameters used in sequence specific assignment in the citrate free state of CitApc with IVFL reverse labeling. The powers for soft pulses are taken at the maximum value.

Spectrum	(H)NH	(H)CANH	(H)(CO)CA(CO)NH
Scans per point	136	24	32
Experimental time / h	15.1	58.8	117.3
Signal/noise	226.17	21.43	N/A
Field	850	850	850
Spinning frequency/Hz	55000	55000	55000
interscan delay /s	1	1	1
Sweep width (t1) / ppm	160 (N)	40 (N)	40(N)
Max indirect evolution (t1) / ms	14.5 (N)	11.0 (N)	9.6 (N)
Sweep width (t2) / ppm	40.3 (H)	30 (C)	84 (C)
Max indirect evolution (t2) / ms	30.0 (H)	9.0 (C)	5.6 (C)
Sweep width (t3) / ppm	N/A	40.3 (H)	40.3 (H)
Max indirect evolution (t3) / ms	N/A	30.0 (H)	30.0 (H)
**Transfer I**	**HN (dipolar)**	**HCA (dipolar)**	**HCA (dipolar)**
^{1}H field / kHz	98.2	100.9	103.6
X field / kHz	38.1	40.1	40.1
Shape	100 - 80 % ramp (H)	85 - 100 % ramp (H)	85 - 100 % ramp (H)
Carrier ^{13}C	N/A	53.7	53.7
Time / ms	1.15	3.5	3.5
**Transfer II**	**HN (dipolar)**	**CAN (dipolar)**	**CACBCA(scalar)**
^{1}H field / kHz	94.5	N/A	N/A
^{13}C field / kHz	N/A	27.4	N/A
^{15}N field / kHz	38.1	26.6	N/A
Carrier ^{13}C	N/A	53.7	53.7 and 48
Shape	100 - 80 % ramp (H)	Tan_ampmod_63_94.land (N)	
Time / ms	0.8	9	6
**Transfer III**		**NH (dipolar)**	**CAN (dipolar)**
^{1}H field / kHz	N/A	98.1	N/A

**Spectrum**	(H)NH	(H)CANH	(H)(CO)CA(CO)NH
^{13}C field / kHz	N/A	N/A	27.4
^{15}N field / kHz	N/A	38.1	26.6
Carrier ^{13}C	N/A	N/A	53.3
Shape	N/A	100 - 80 % ramp (H)	Tan_ampmod_63_94.land (N)
Time / ms	N/A	0.85	9
**Transfer IV**			**NH (dipolar)**
^{1}H field / kHz	N/A	N/A	97.2
^{13}C field / kHz		N/A	N/A
^{15}N field / kHz	N/A	N/A	38.1
Carrier ^{13}C	N/A	N/A	N/A
Shape	N/A	N/A	100 - 80 % ramp (H)
Time / ms	N/A	N/A	0.85

Supplementary Table 5. NMR experimental parameters used in sequence specific assignment in the citrate bound state of CitApc with IVFL reverse labeling. The powers for soft pulses are taken at the maximum value.

**Spectrum**	(H)NH	(H)CANH	(H)(CO)CA(CO)NH	(H)(CA)CB(CA)NH	(H)(CA)CB(CA)(CO)NH
Scans per point	80	16	64	32	72
Experimental time / h	6.2	85.3	153.5	158.4	337.9
Signal/noise	181.46	21.65	10.86	N/A	N/A
Field	850	850	850	850	850
Spinning frequency/Hz	55000	55000	55000	55000	55000
interscan delay /s	1	2	1	1	1
Sweep width (t1) / ppm	120 (N)	36 (N)	36 (N)	36(N)	34(N)
Max indirect evolution (t1) / ms	13.5 (N)	16.1 (N)	14.5 (N)	14.5 (N)	15.0 (N)
Sweep width (t2) / ppm	40.8 (H)	32 (C)	32 (C)	70.0 (C)	70 (C)
Max indirect evolution (t2) / ms	29.5 (H)	7.0 (C)	7.0 (C)	6.6 (C)	6.4 (C)
Sweep width (t3) / ppm	N/A	58.8 (H)	58.8 (H)	58.8 (H)	58.8 (H)
Max indirect evolution (t3) / ms	N/A	20.5 (H)	20.5 (H)	20.5 (H)	20.5 (H)
**Transfer I**	**HN (dipolar)**	**HCA (dipolar)**	**HCO (dipolar)**	**HCA (dipolar)**	**HCA (dipolar)**
^{1}H field / kHz	96	92.9	82.6	90.7	87.3
X field / kHz	38.1	41	41	41	37.7
Shape	100 - 80 % ramp (H)	85 - 100 % ramp (H)	85 - 100 % ramp (H)	85 - 100 % ramp (H)	85 - 100 % ramp (H)
Carrier ^{13}C		53.7	173.3	53.7	53.7
Time / ms	1	6	6	5.5	4
**Transfer II**	**HN (dipolar)**	**CAN (dipolar)**	**COCA (scalar)**	**CACBCA(scalar)**	**CACBCA(scalar)**
^{1}H field / kHz	95.4	N/A	N/A	N/A	N/A
^{13}C field / kHz	N/A	36	N/A	N/A	N/A

Spectrum	(H)NH	(H)CANH	(H)(CO)CA(CO)NH	(H)(CA)CB(CA)NH	(H)(CA)CB(CA)(CO)NH
^{15}N field / kHz	38.1	28.4	N/A	N/A	N/A
Carrier ^{13}C	N/A	53.7	173.3 and 53.3	53.7 and 48	53.7 and 48
Shape	100 - 80 % ramp (H)	Tan_ampmod_63_94.land (N)		N/A	N/A
Time / ms	0.8	11	15.6	18.4	18.4
**Transfer III**		**NH (dipolar)**	**CON (dipolar)**	**CAN (dipolar)**	**COCA (scalar)**
^{1}H field / kHz	N/A	95.4	N/A	N/A	N/A
^{13}C field / kHz	N/A	N/A	35.4	36	N/A
^{15}N field / kHz	N/A	38.1	31	28.9	N/A
Carrier ^{13}C	N/A	N/A	173.3	53.3	173.3 and 53.3
Shape	N/A	100 - 80 % ramp (H)	Tan_ampmod_63_94.land (N)	Tan_ampmod_63_94.land (N)	
Time / ms	N/A	1.15	11	11	15.6
**Transfer IV**			**NH (dipolar)**	**NH (dipolar)**	**CON (dipolar)**
^{1}H field / kHz	N/A	N/A	92.2	89.6	N/A
^{13}C field / kHz	N/A	N/A	N/A	N/A	33.5
^{15}N field / kHz	N/A	N/A	38.1	38.1	30.3
Carrier ^{13}C	N/A	N/A	N/A	N/A	173.3
Shape	N/A	N/A	100 - 80 % ramp (H)	100 - 80 % ramp (H)	Tan_ampmod_63_94.land (N)
Time / ms	N/A	N/A	1.15	0.8	13
**Transfer V**					**NH (dipolar)**
^{1}H field / kHz	N/A	N/A	N/A	N/A	88.7
^{13}C field / kHz	N/A	N/A	N/A	N/A	N/A
^{15}N field / kHz	N/A	N/A	N/A	N/A	38.1
Carrier ^{13}C	N/A	N/A	N/A	N/A	N/A
Shape	N/A	N/A	N/A	N/A	100 - 80 % ramp (H)
Time / ms	N/A	N/A	0.85	N/A	0.85

Supplementary Table 6. Data collection and refinement statistics Gt PASp-Citrate complex

	Gt PASp-Citrate*
**Data collection**	
Space group	$P2_1$
Cell dimensions	
a, *b*, *c* (Å)	64.21, 101.14, 77.88
α, β, γ (°)	90, 90, 90
Wavelength (Å)	1.0
Resolution (Å)	48.79-1.70 (1.73-1.70)**
R_{sym}	5.8 (54.2)
$I / \sigma I$	16.47 (2.71)
Completeness (%)	99.9 (99.5)
Redundancy	6.85 (6.86)
**Refinement**	
Resolution (Å)	48.79-1.70
No. reflections	105,114
R_{work} / R_{free}	17.31 / 20.96
No. atoms***	
Protein	1029
Ligand/ion	
citrate	8
sodium	1
Water	726
B-factors	
Protein	25.57
Ligand/ion	
citrate	20.62
sodium	33.29
Water	35.9
R.m.s. deviations	
Bond lengths (Å)	0.011
Bond angles (°)	1.65

*Structure solved from single crystal. **Values in parentheses are for highest-resolution shell.***Number of atoms in asymmetric unit.

Supplementary Table 7. Data collection and refinement statistics Gt PASp-R93A

	Gt PASP-R93A*
**Data collection**	
Space group	$C222_1$
Cell dimensions	
a, *b*, *c* (Å)	60.14, 120.05, 152.14
α, β, γ (°)	90, 90, 90
Wavelength (Å)	1.0
Resolution (Å)**	47.12-1.61 (1.64-1.61)
R_{sym}	7.5 (68.6)
$I / \sigma I$	16.33 (1.27)
Completeness (%)	99.1 (93.3)
Redundancy	12.94 (10.44)
**Refinement**	
Resolution (Å)	47.12 -1.61
No. reflections	70,580
R_{work} / R_{free}	19.1 / 22.3
No. atoms***	
Protein	500
Ligand/ion	
CXS	4
sulfate	3
glycerol	2
Water	221
B-factors	
Protein	33.77
Ligand/ion	
CXS****	47.69
Sulfate	43.24
glycerol	44.66
Water	41.56
R.m.s. deviations	
Bond lengths (Å)	0.007
Bond angles (°)	0.975

*Structure solved from single crystal. **Values in parentheses are for highest-resolution shell.***Number of atoms in asymmetric unit.****3-Cyclohexyl-1-propylsulfonic acid.

Supplementary Table 8. Data collection, phasing and refinement statistics for Gt PASc-N288D

		Gt PASc-N288D*	
**Data collection**			
Space group		$P2_12_12_1$	
Cell dimensions			
a, *b*, *c* (Å)		48.94, 49.31, 92.19	
α, β, γ (°)		90, 90, 90	
	Peak	*Inflection*	*Remote*
Wavelength	0.97957	0.9800	0.97188
Resolution (Å)**	43.51-2.13 (2.17-2.13)	46.35-2.50 (2.56-2.50)	43.51-2.10 (2.15-2.10)
R_{sym}	7.4 (46.5)	6.9 (63.3)	3.7 (34.4)
$I / \sigma I$	15.75 (2.76)	21.26 (3.67)	20.96 (3.0)
Completeness (%)	93.1 (83.9)	99.5 (100)	92.5 (80.1)
Redundancy	3.17 (2.6)	12.84 (13.39)	3.13 (2.51)
**Refinement**			
Resolution (Å)		43.47-2.10	
No. reflections	12,062	8245	12,630
R_{work} / R_{free}		19.5 / 23.3	
No. atoms***			
Protein		219	
Ligand/ion			
magnesium		1	
Water		33	
B-factors			
Protein		46.72	
Ligand/ion			
magnesium		50.77	
Water		42.53	
R.m.s deviations			
Bond lengths (Å)		0.009	
Bond angles (°)		1.614	

*Structure solved from single crystal. **Values in parentheses are for highest-resolution shell.***Number of atoms in asymmetric unit.

Supplementary Table 9. Parameters extracted from CEST profile fitting of the WT PASc domain.

	exchange rates				
Kab [s-1]	17.731 +/- 5.147				
Kba [s-1]	450.471 +/- 94.875				
residue	Peak position [ppm]	CS difference [ppm]	R1 [s-1]	R2a [s-1]	R2b [s-1]
E209	116.9445 +/- 0.0709	3.5118 +/- 0.2071	1.1831 +/- 0.0213	87.1442 +/- 6.2674	38.4808 +/- 75.2772
E215	114.7406 +/- 0.0415	4.5057 +/- 0.2311	0.4289 +/- 0.0215	62.1840 +/- 4.4704	74.8535 +/- 79.2006

Supplementary Table 10. Parameters extracted from CEST profile fitting of the N288D mutant PASc domain.

	exchange rates				
Kab [s-1]	2.409 +/- 0.281				
Kba [s-1]	70.442 +/- 13.434				
residue	Peak position [ppm]	CS difference [ppm]	R1 [s-1]	R2a [s-1]	R2b [s-1]
A212	120.4246 +/- 0.0549	2.8159 +/- 0.1641	0.4981 +/- 0.0163	34.2863 +/- 2.2802	38.8838 +/- 56.3272
L214	117.8093 +/- 0.0378	5.2163 +/- 0.1215	0.3986 +/- 0.0157	31.4040 +/- 2.0766	142.6128 +/- 33.4526
E215	113.6648 +/- 0.0406	5.2638 +/- 0.1194	0.5784 +/- 0.0218	36.4141 +/- 2.4877	254.6045 +/- 60.6037
I217	114.0103 +/- 0.0362	4.9695 +/- 0.1101	0.5383 +/- 0.0147	36.4148 +/- 2.6105	126.8150 +/- 24.4825

Supplementary Table 11. Intensity of the ^{19}F CODEX experiment of the CF3-N308C CitA PASpc in the citrate free state and the intensities from their respective reference experiments.

mixing time [ms]	S	S0	Noise
250	42.28	49.41	1
300	7.67	9.61	1
500	34.29	34.52	1

Supplementary Table 12. Intensity of the ^{19}F CODEX experiment of the CF3-N308C CitA PASpc in the citrate bound state and the intensities from their respective reference experiments.

mixing time [ms]	S	S0	Noise
20	66.16	70.41	1
50	19.62	19.09	1
100	27.95	30.86	1
200	9.72	13.59	1
250	11.33	17.33	1
300	6.66	11.71	1
400	12.17	23.96	1
500	9.58	18.66	1
