# Supplementary material for: Mechanism of sensor kinase CitA transmembrane signaling
Source: Nat Commun. 2025 Jan 22;16:935. doi: 10.1038/s41467-024-55671-3 (PMC11754779; doi:10.1038/s41467-024-55671-3)
Supplement: Supplementary file 2 — Description of Additional Supplementary Files [file 41467_2024_55671_MOESM2_ESM.pdf]

Description of Additional Supplementary Files

File Name: Supplementary Data 1
Description: Proton-1, carbon-13 and nitrogen-15 chemical shifts of CitApc in the citrate bound state in ppm.

File Name: Supplementary Data 2
Description: Proton-1, carbon-13 and nitrogen-15 chemical shifts of CitApc in the citrate free state in ppm.
